# Epidemiology of immune-mediated thrombotic thrombocytopenic purpura in Germany

**DOI:** 10.1016/j.rpth.2026.106646

**Published:** 2026-05-29

**Authors:** Ulf Schönermarck, Florian Wehrmann, Juliane Schneider, Michael Fischereder, Anke von Bergwelt-Baildon

**Affiliations:** Division of Nephrology, Department of Medicine IV, LMU University Hospital, LMU Munich, Munich, Germany

Dear Editor:

In their retrospective population-based cohort study, Scharenberg et al [1] used nationwide inpatient data to assess the incidence and outcome of immune-mediated thrombotic thrombocytopenic purpura (iTTP) in Germany between 2011 and 2021. The reported annual incidence of iTTP episodes between 2.36 and 3.48 per million inhabitants fits well within published data of 1.5 and 6.0 cases per million worldwide [2]. A recent analysis in Germany found an annual projected incidence of 2.10 acute iTTP episodes per million inhabitants (95% CI, 1.60-2.58) in the years 2014-2016 [3].

However, the reported complication rates are unexpectedly high: 12.8% in-hospital mortality, 33.5% multiorgan failure, and 25.5% kidney replacement therapy [1]. Large registry data and recent randomized studies reported mortality rates of less than 10% [[4], [5], [6]]. While mild episodes of acute kidney injury (AKI) are common and rapidly resolving, severe AKI with need for kidney replacement therapy can occur but are reported usually in less than 5% to 10% of patients [[4], [5], [6], [7]].

To detect inpatient cases with acute episodes of iTTP, the ICD-10-GM (International Classification of Diseases, Tenth Revision, German Modification) code M31.1 was used in combination with at least 1 documented plasma exchange. However, the ICD-10-GM code M31.1 describes the group of thrombotic microangiopathies (TMAs) including not only iTTP but also a variety of other TMA forms with different pathophysiologies. Plasma exchange is often used as first-line therapy awaiting the results of ADAMTS-13 (a disintegrin and metalloproteinase with thrombospondin motifs 13) measurement, and kidney involvement is more common in other TMA forms including (atypical) hemolytic–uremic syndrome.

On the other hand, the authors excluded patients with the diagnosis of systemic lupus erythematosus (ICD-10-GM code M32). However, patients with iTTP exhibit a higher prevalence of concomitant autoimmune diseases, and the association between systemic lupus erythematosus and iTTP is well described, occurring in 6% to 7% of patients [8].

In conclusion, the use of ICD-10-GM code M31.1, which is not exclusive for iTTP, might overestimate the incidence and could explain the higher complication rates in this study. In our clinical practice, episodes of AKI in iTTP are usually mild and rapidly resolving. Severe AKI with requirement of kidney replacement therapy or prolonged kidney function impairment should prompt the search for underlying diseases other than iTTP and rigorous performance of a kidney biopsy.
